# Integrated analysis of mRNA, microRNA and protein in systemic lupus erythematosus-specific induced pluripotent stem cells from urine

**DOI:** 10.1186/s12864-016-2809-9

**Published:** 2016-07-11

**Authors:** Donge Tang, Yuyu Chen, Huiyan He, Jianrong Huang, Wenbiao Chen, Wujian Peng, Qianjin Lu, Yong Dai

**Affiliations:** Key Laboratory of Functional Protein Research of Guangdong Higher Education Institutes, Institute of Life and Health Engineering, College of Life Science and Technology, Jinan University, Guangzhou, 510632 People’s Republic of China; Clinical Medical Research Center, The Second Clinical Medical College of Jinan University (Shenzhen People’s Hospital), Shenzhen, Guangdong 518020 People’s Republic of China; Department of Dermatology, Second Xiangya Hospital, Central South University, Hunan Key Laboratory of Medical Epigenomics, Changsha, Hunan 410011 People’s Republic of China

**Keywords:** Systemic lupus erythematosus (SLE), Induced pluripotent stem cells (iPSCs), Proteins, mRNA, microRNA

## Abstract

**Background:**

In clinical practice, it is difficult to monitor the repeating relapse in patients who have been suffering from systemic lupus erythematosus (SLE). The underlying etiology remains largely unknown.

**Methods:**

Aiming to understand the pathogenesis of SLE, a detailed study was conducted. Renal tubular cells–derived iPSCs were successfully obtained from the urine of SLE patients and healthy controls. With the purpose to identify simultaneous expression profiling of microRNA, mRNA and protein, Illumina HiSeq™ 2000 System and iTRAQ-coupled 2D LC-MS/MS analysis were utilized in systemic lupus erythematosus-specific induced pluripotent stem cells (SLE-iPSCs) and normal control-iPSCs (NC-iPSCs). The integration of multiple profiling datasets was realized since it could facilitate the identification of non-seed miRNA targets, as well as differentially expressed mRNAs and proteins.

**Results:**

For this study, profiling datasets of 1099 differentially expressed mRNAs, 223 differentially expressed microRNAs and 94 differentially expressed proteins were integrated. In order to investigate the influence of miRNA on the processes of regulating mRNAs and proteins’ levels, potential targets of differentially expressed mRNAs and proteins were predicted using miRanda, TargetScan and Pictar. Multiple profiling datasets were integrated to facilitate the identification of miRNA targets, as well as differentially expressed mRNAs and proteins. Through gene ontology (GO) analysis of differentially expressed mRNAs and proteins, biological processes that drive proliferation were identified, such as mRNA processing and translation. Western blot and Q-PCR confirmed AK4 protein and mRNA up-regulation. The findings also showed that TAGLN’s protein and mRNA level were down-regulated in SLE-iPSCs, both miR-371a-5p and let-7a-5p in SLE-iPSC were down-regulated and verified using Q-PCR. The up-regulation of AK4 involved in nucleotide biosynthesis suggested a general acceleration of anabolic metabolism induced by down-regulated miR-371a-5p, which might contribute to SLE.

**Conclusion:**

Based on high throughput analysis, integrated miRNA, mRNA, and protein expression data were generated. Differentially expressed dates were also adopted in conjunction with in-silico tools to identify potential candidates for SLE-iPSCs. Representative miRNA, mRNA and proteins were verified. It was also expected that the knowledge gained from this study can be applied to assess the usefulness of pathogenesis and novel biomarker candidates of SLE, which may develop a new way for SLE diagnosis.

**Electronic supplementary material:**

The online version of this article (doi:10.1186/s12864-016-2809-9) contains supplementary material, which is available to authorized users.

## Background

Systemic lupus erythematosus (SLE) is the prototype of complex autoimmune disease characterized by the production of autoantibodies which results in widespread immunologic abnormalities and immune complex formation [1]. The patients can present variable manifestations and the nature courses are alternately remissions and relapses. Till now, although a lot of related researches have been undertaken [2], SLE patients have no effective cures, whose treatments are often based upon long-term broad-spectrum immune suppressive regimes in the current therapeutic management. It becomes a major public health problem.

The exact mechanism involved in SLE is needed to understand. a lot of work has been done in searching for biomarkers, from the aspects of DNA, mRNA and protein, expecting to illustrate the mechanism of SLE and find ideal biomarkers for diagnosis and as a precaution to SLE [3–5]. However, the underlying mechanism and pathogenesis of SLE are still far away from understanding. Novel methods should be looked into in this area.

As is known to all, the possibility of reprograming somatic cells to induced pluripotent stem cells (iPSCs) offers an opportunity to generate pluripotent patient-specific cell lines, which can be beneficial for studying pathogenesis of model human diseases [6]. Also, these iPSCs lines are powerful tools for recapitulating disease conditions and thus better understanding the underlying mechanisms and pathogenesis of specific diseases [6]. iPSCs can be derived from immune cells as equally as they can differentiate into specific immune cell types for modeling diseases or clinical immunotherapy [6]. So far, generations of iPSCs from urine, fibroblasts and keratinocytes of disease patients have been reported [7, 8]. On this basis, renal tubular cells-derived iPSCs were successfully acquired from urine of SLE patients to study SLE pathogenesis [9].

Currently, extensive researches, including those involving system-wide genomic and transcriptomic approaches, have been conducted to characterize iPSCs [10]. However, molecular mechanisms remain insufficiently understood. At the molecular level, iPSCs are more variable than embryonic stem cells (ESCs) [11]. It is imperative that the compendium of differences described between iPSCs and ESCs be considered as further evidence for the fact that the reprogramming process requires a wide variety of molecular changes. Besides, cells can distinguish bona fide iPSCs from partial reprogrammed cells during an earlier stage [11]. Therefore, systematic deciphering of genetic or epigenetic alternations would help to identify hotspots in iPSCs with consistent genetic background [12].

Briefly, renal tubular cells–derived iPSCs were successfully obtained from urine of SLE patients and healthy controls to understand the pathogenesis of SLE. For the integration of multiple profiling datasets, simultaneous expression profiling of microRNA, mRNA and protein in SLE-iPSCs and NC-iPSCs were identified with Illumina HiSeq™ 2000 System and iTRAQ-coupled 2D LC-MS/MS analysis.

## Results

### mRNA, protein, miRNA expression profiles

**Fig. 1:**
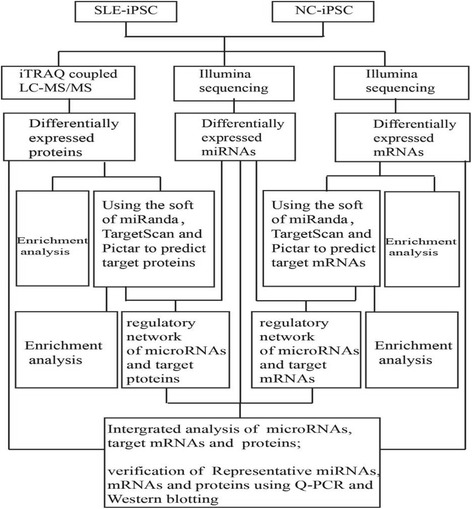
Data analysis overview. Stage 1: SLE-iPSCs and NC-iPSCs were used for the extraction of protein and RNA. Stage 2: Proteins were identified with iTRAQ-coupled LC–MS/MS; the libraries of mRNA and miRNA were constructed by using the Illumina TruSeq RNA Sample Prep Kit v2-Set A and TruSeq Small RNA Sample Prep Kit Set A, respectively, and then sequenced by using the Illumina HiSeq™ 2000 System. Stage 3: Protein dates were submitted to the ProteinPilot analysis software for peptide identification and quantification, subsequently, differentially expressed proteins were identified and quantified. Gene expressions based on sequencing were measured by RPKM values, ‘FDR(false discovery rate) ≤0.001 and the absolute value of log2-Ratio ≥1’ as the threshold. Differentially expressed mRNAs and miRNAs were obtained. Stage 4: GO enrichment analysis facilitated the mappings of all differentially expressed proteins, mRNAs and miRNAs to GO terms in the database (http: //www. geneontology.org/). With PicTar, miRanda v5, TargetScan 5.1, differentially expressed target proteins and mRNAs were predicted. Stage 5: Differentially expressed proteins and mRNAs were classified according to the GO database. With Cytoscape software,the regulation network of microRNA-target protein and microRNA-target mRNA were analyzed. Stage 6: An integrated analysis of microRNA, target mRNAs and proteins was carried out using Cytoscape software. Representative miRNA, mRNA and proteins were verified using Q-PCR and western blotting

In order to comprehensively reveal mRNA, microRNA and protein interactions, we obtained high quality, complete information and estimated the expression levels of mRNA, miRNA and protein between the SLE-iPSCs and control-iPSCs (Fig. 1).

For the mRNA expression profiling, *P*-value ≤ 0.05 and FDR ≤ 0.001 were set as threshold value, 4,254 genes were detected to have at least two-fold differences between SLE-iPSCs and control-iPSCs, The genes numbers of 2,856 and 1,398 respectively represent the higher and lower abundances of more than two fold compared with control-iPSCs. Among 4,254 genes, 1,099 differentially expressed ones built an interaction network, with 744 genes up-regulated and 355 down-regulated (Additional file 1: Table S1).

Compared with the control group, it was found in the microRNA libraries that 223 miRNAs were expressed at the level of significant difference, with 126 up-regulated miRNAs and 97 down-regulated ones in SLE-iPSCs (Additional file 2: Table S2).

In the protein expression profiling, the identification and quantification of 2,305 proteins in SLE-iPSCs and control-iPSCs were performed with iTRAQ technique. Given a confidence level of 95 %, 1.5-fold was set as the cut-off criterion for up-regulated and down-regulated proteins. Under these criteria, a total of 207 proteins were classified as differentially expressed ones between SLE-iPSCs and control-iPSCs, with 55 showing increased abundance and the remaining 152 decreased abundance (Additional file 3, Additional file 4: Table S3,4).

### MicroRNA-target genes regulation network

**Fig. 2:**
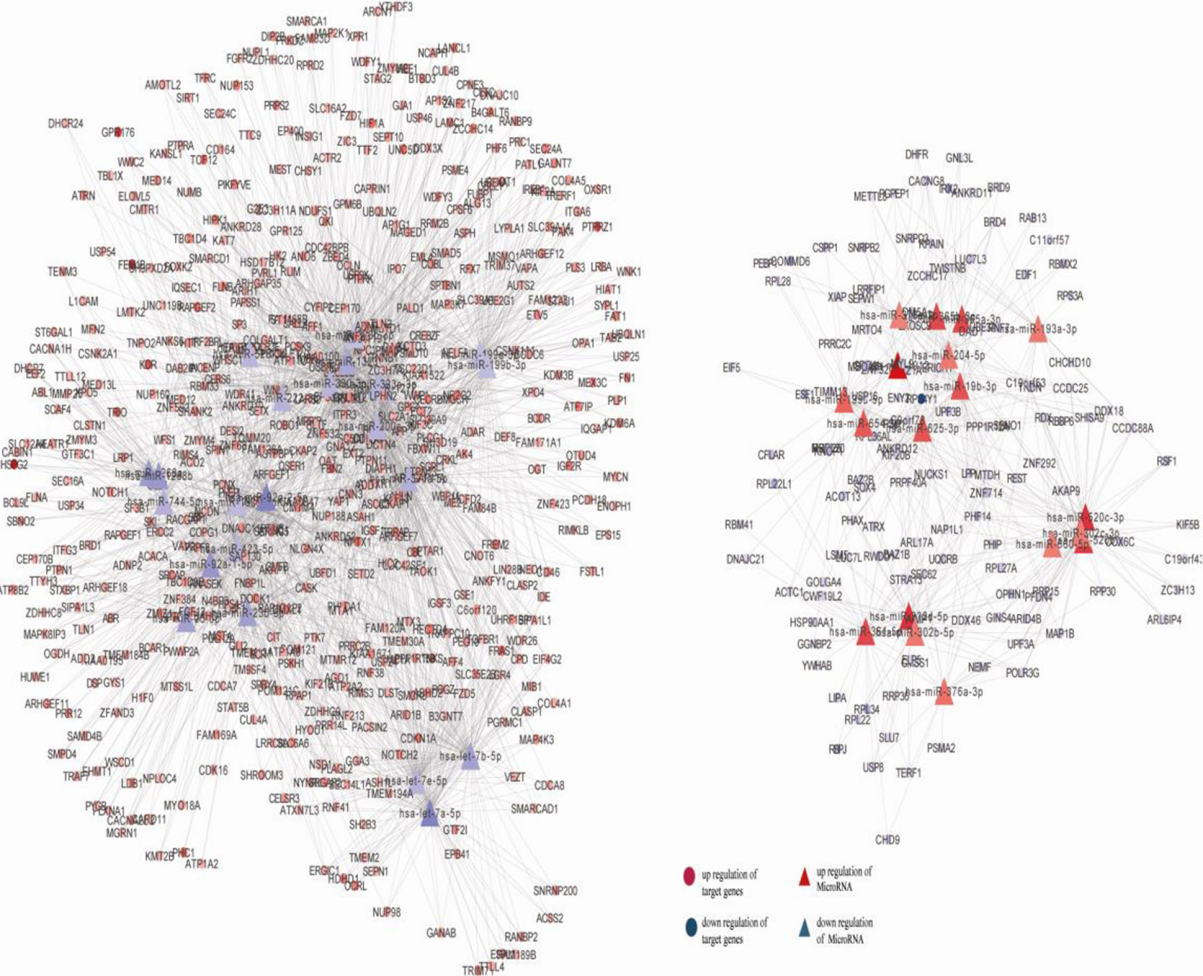
MicroRNA-target network. Circles represent miRNA-target mRNAs, and triangles signify mRNA. The red nodes denote up-regulation, while the blue nodes symbolize down-regulation. The lines stand for coherent miRNA -target genes interaction pairs

**Fig. 3:**
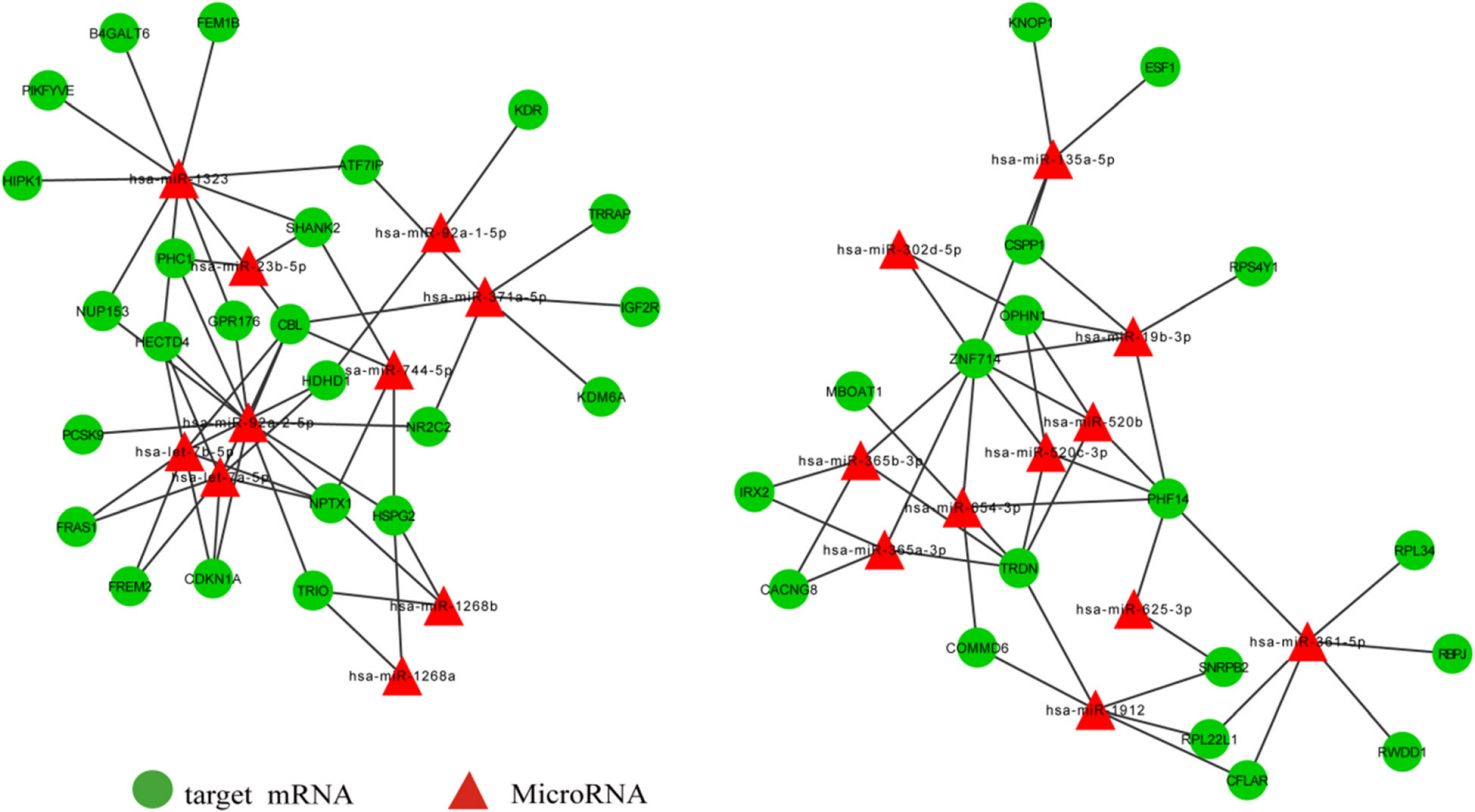
microRNA-target mRNA sub-network. The mapping was constituted by 43 down-regulated target mRNAs and 21 up-regulated miRNAs. The green circles represent down-regulated target mRNAs, and the red triangles denote up-regulated miRNAs. The lines stand for coherent miRNAs-target mRNAs interaction pairs. A single miRNA can target multiple proteins, while a single protein can be targeted by several miRNAs. Multiple miRNAs can cooperatively repress a range of targets

With integrated mRNA and microRNA transcriptome, the expression change trend of miRNA target mRNAs were focused on. Then, a systematically investigation was conducted on the potential functional correlations among microRNA target mRNAs. Cytoscape software was used to construct the regulation network of differentially expressed mRNAs. Their microRNA-target mRNAs were predicted by using miRanda, TargetScan and Pictar. If the target mRNAs were successfully predicted at least with two kinds of software at the same time, they would be considered to be reliable. MicroRNA-target mRNAs regulation network was shown in Figs. 2, 3. It can be seen that microRNA and target genes were mutually cross-regulated.

**Fig. 4:**
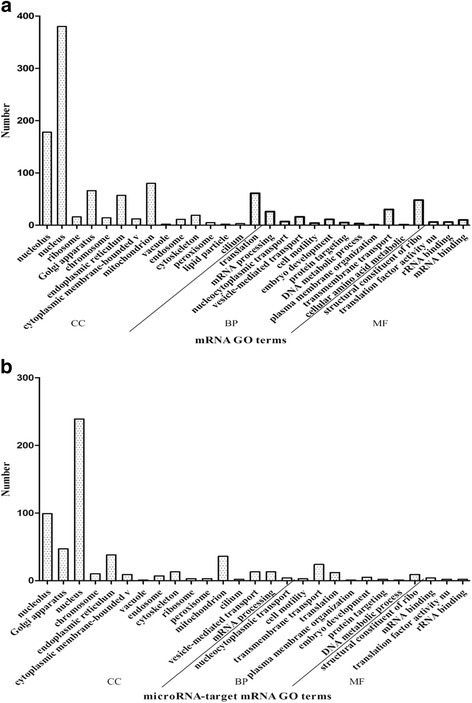
Gene ontology (GO) analysis. **a** GO analysis of differently expressed mRNA with p < 0.05, **b** GO analysis of differentially regulated target mRNA. The horizontal axis indicates the names of the clusters in cellular component (CC), biological process (BP) and molecular function (MF), respectively. The vertical axis displays the numbers of targets. The GO terms were sorted by the enrichment P-value, in an ascending order of p-value from bottom to top

A functional analysis was conducted based on Gene Ontology. Some gene ontologies of biological processes, molecular functions and cellular components were selected, which were enriched by the features of the transcriptomic and proteomic datasets with *p* value < 0.05. In the study, GO enrichment analysis was performed through functional annotation clustering of microRNA and target genes, respectively. Totally, 11 biological processes (BP), 4 molecular functions (MF) and 14 cellular components (CC) GO terms were enriched in target genes. 10 BP, 4 MF and 13 CC GO terms in microRNA are shown in Fig. 4 a–b, respectively. BP includes translation, mRNA processing, nucleocytoplasmic transportation, vesicle-mediated transportation, cell motility and so on. MF involves structural constituent of ribosome, translation factor activity, nucleic acid binding, rRNA binding, mRNA binding. CC contains nucleolus nucleus, ribosome, Golgi apparatus, chromosome, endoplasmic reticulum, cytoplasmic membrane-bounded vesicle, mitochondrion, cytoplasmic membrane-bounded vesicle and so on. According to the biological process, the microRNA and target genes are mostly involved in BP GO terms, structural constituent of ribosome of MF GO term and nucleus of CC GO terms.

Generally, microRNAs are classified as a class of small non-coding RNAs that bind complementary sequences in target mRNAs to specifically regulate gene expression through either mRNA degradation or translational inhibition [13]. If a target gene is down-regulated, it suggests that effective activity of miRNA is enhanced under the treatment, while an up-regulation of a target gene indicates a decreased activity of the corresponding miRNAs. Consequently, a miRNA-mRNA interaction pair means anti-regulation of a miRNA and a corresponding mRNA [14, 15].

### MicroRNA-target protein regulation network

**Fig. 5:**
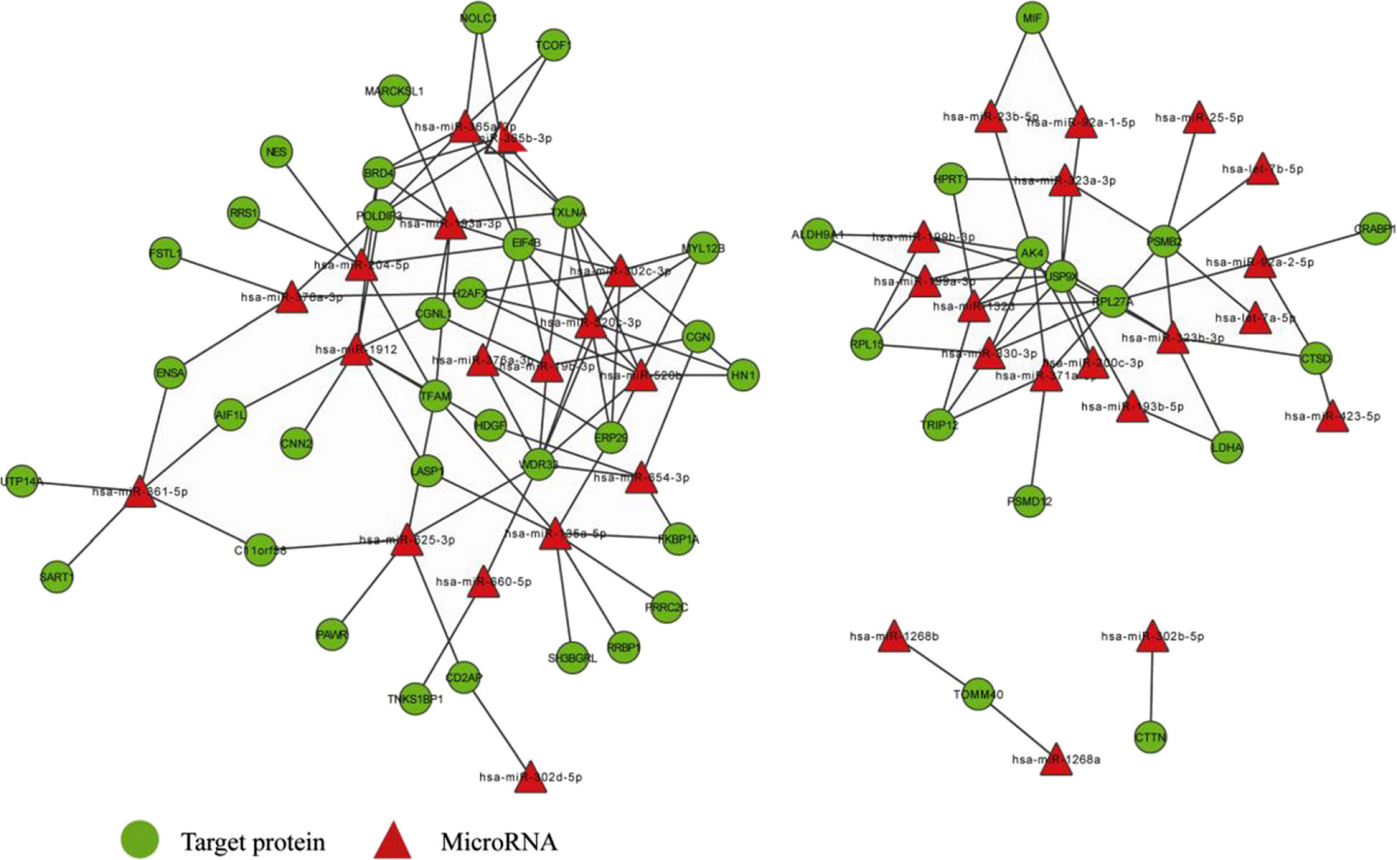
microRNA-target protein network. The mapping is composed of 48 differentially regulated target proteins and 36 differentially expressed miRNAs. Green circles represent down- regulated target proteins, and red triangles signify up-regulated miRNAs. The lines stand for coherent miRNAs-target proteins interaction pairs. A single miRNA can target multiple proteins, while a single protein can be targeted by several miRNAs. Multiple miRNAs can cooperatively repress a range of targets

In this study, a comparative proteome survey was performed on the SLE-iPSC and control-iPSC using iTRAQ technique. The identification and quantification of differentially expressed proteins were realized. In order to investigate the potential functional correlations among microRNA target proteins, an integrated systematic analysis was made on microRNA and protein data. In addition, Cytoscape software was applied to construct the regulation network of microRNA and target protein. Given a 95 % confidence level, the interaction network of 94 proteins in 207 differentially expressed proteins were classified (Fig. 5); 37 MicroRNAs which regulated 49 target proteins were predicted. MicroRNA-target proteins regulation network is shown in Fig. 5, which manifests that microRNA- target protein were mutually cross-regulated.

**Fig. 6:**
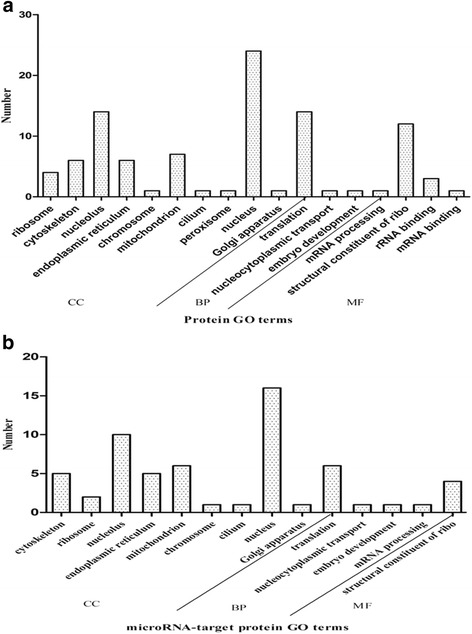
Gene ontology (GO) analysis. **a** GO analysis of differently expressed proteins with p < 0.05, **b** GO analysis of differentially regulated target proteins. The horizontal axis indicates the names of the clusters in cellular component (CC), biological process (BP) and molecular function (MF), respectively. The vertical axis displays the numbers of targets. The GO terms were sorted by the enrichment P-value, in an ascending order of p-value from bottom to top

GO enrichment analyses of microRNA and target proteins were performed, respectively. Totally, 4 BP, 3 MF and 10 CC GO terms were enriched in target proteins. 4 BP, 1MF and 9 CC GO terms in microRNA are respectively shown in Fig. 6a–b. BP includes translation, nucleocytoplasmic transport, embryo development and mRNA processing; MF contains structural constituents of ribosome; CC involves nucleolusnucleus, ribosome, endoplasmic reticulum, mitochondrion, cytoskeleton and so on. According to biological process, the microRNA-target proteins are mostly involved in the translation of BP GO terms, structural constituent of ribosome of MF GO term and nucleus of CC GO terms.

### mRNA-protein regulation network

**Fig. 7:**
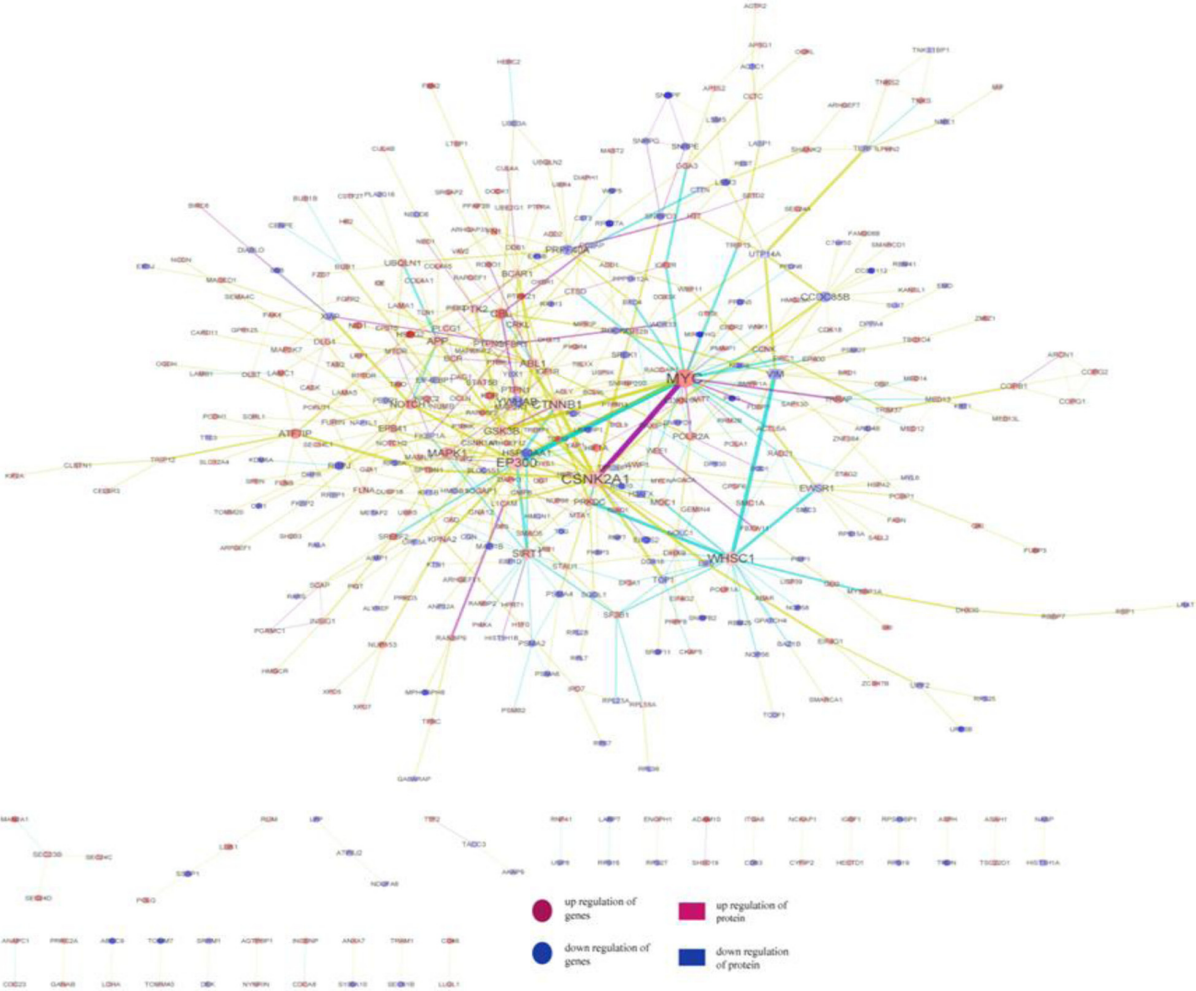
Differentially expressed mRNA-Proteomic mapping. Differentially expressed mRNA- Proteomic regulation network was constructed. Tetragons represent protein, and circles signify mRNA. The red nodes stand for up-regulation, and the blue nodes down-regulation. The red lines denote experiment support, yellow lines interaction from PubMed, and blue lines interaction from database

For the purpose of identifying the correlations between mRNA and protein expressions, the expression change trend of transcriptomic and proteomic profiles were focused on. 1099 differentially expressed mRNAs and proteins were found to be correlated in the mRNA-protein library (Fig. 7).

A functional analysis was conducted through the KEGG pathway. Given a cut-off criterion of Q-value < 0.05, 1109 differentially-expressed mRNAs were involved in 235 pathways, and 99 differentially expressed proteins in 65 pathways (Additional file 5, Additional file 6: Table S5,6). According to the enrichment analysis, a few important pathways were significantly enriched in response to SLE-iPSCs. It was quite evident that mRNAs and proteins were both involved in ribosome, spliceosome, RNA transport, lysine degradation and so on.

**Fig. 8:**
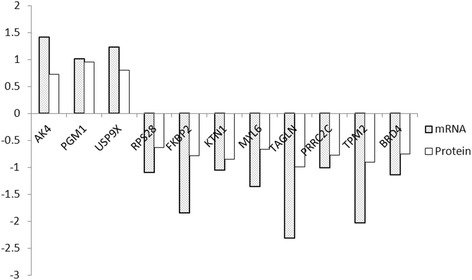
A direct correspondence between mRNA transcripts and generated protein expressions was revealed

**Fig. 9:**
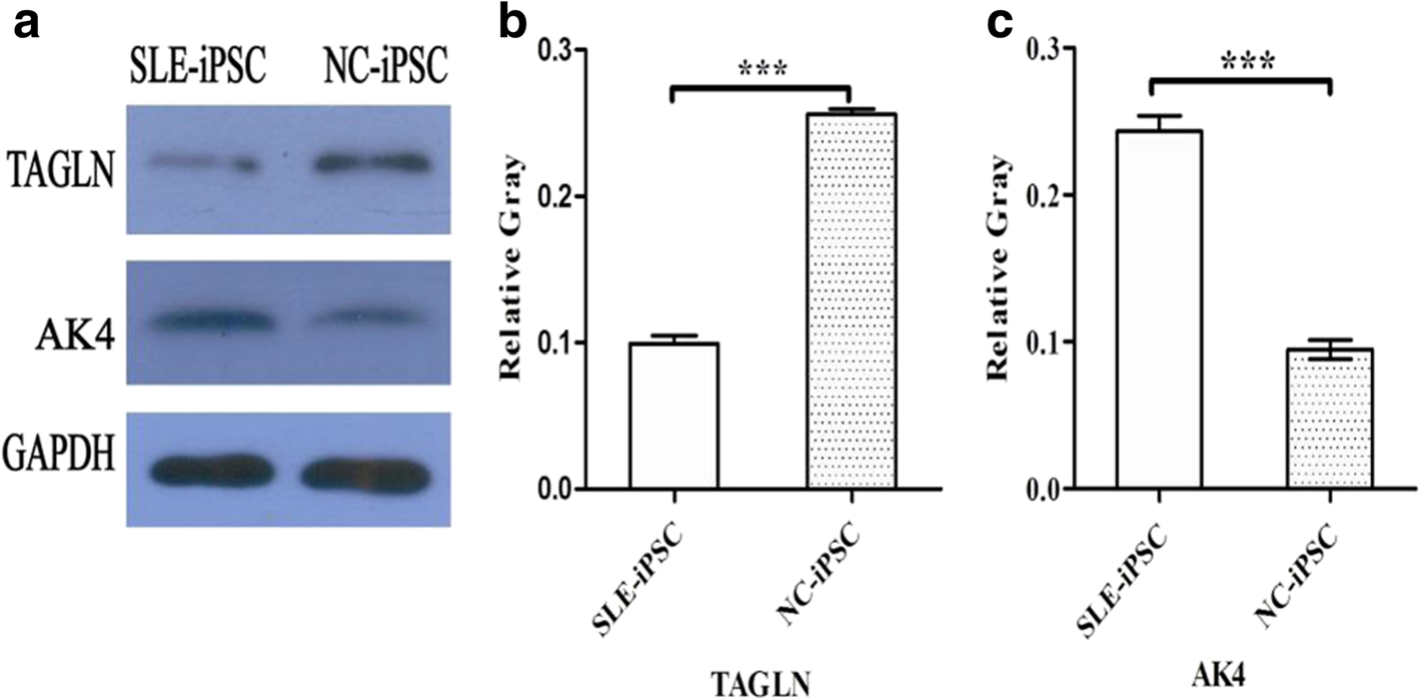
Representative confirmations of relative gene expression in SLE-iPSCs and NC-iPSCs. **a** Representative TAGLN and AK4 were directly detected through Western blotting using anti- TAGLN and AK4 antibodies, respectively. GAPDH was set as internal control with appropriate antibodies. **b**, **c** Relative quantification of immunoblots. The vertical axes indicate the relative gray of TAGLN /GAPDH (B) and AK4/GAPDH **c**, respectively. The horizontal axes display the groups of SLE-iPSCs and NC-iPSCs. Values of SD ± mean were plotted. Asterisk indicates the value compared with that of control group with * *P* < 0.05

**Fig. 10:**
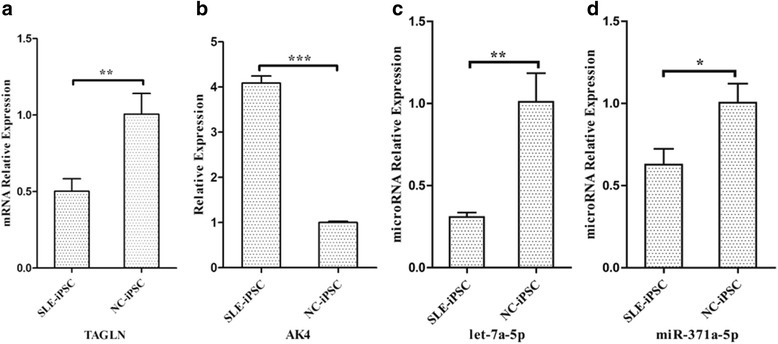
Relative mRNA and miRNA levels in SLE-iPSCs and NC-iPSCs. The mRNA levels of TAGLN **a** and AK4 **b**, as well as the miRNA level of let-7a-5p **c** and miR-371a-5p **d** were used for Q-PCR experiments. The 18S rRNA level was set as the internal control of mRNA, and the U6 SnRNA level as that of miRNA. The data were expressed as means ± SEM of three independent experiments. Asterisk indicates value compared with that of control group with * *P* < 0.05

Based on the central dogma, it was generally assumed that there was a direct correspondence between mRNA transcripts and generated protein expressions. In recent years, various studies have demonstrated the role of RNA-binding proteins (RBPs) [16]. On the basis of mRNA-protein correlation, it was found that 11 proteins and 11 mRNAs were differentially expressed consistently (3 up-regulated and 8 down-regulated, Fig. 8). To further validate the expression data, 2 differentially expressed mRNAs and proteins were randomly chosen and verified with Q-PCR and western blotting (Figs. 9, 10).

The common KEGG pathway of differentially expressed proteins and mRNAs were analyzed using GenMAPP v2.1. KEGG enrichment analyses showed that proteins and mRNA significantly participated in ECM-receptor interaction, fatty acid metabolism, ribosome, spliceosome, RNA transport and so on(Additional file 5, Additional file 6: Table S5,6).

### mRNA-protein-microRNA regulation network

**Fig. 11:**
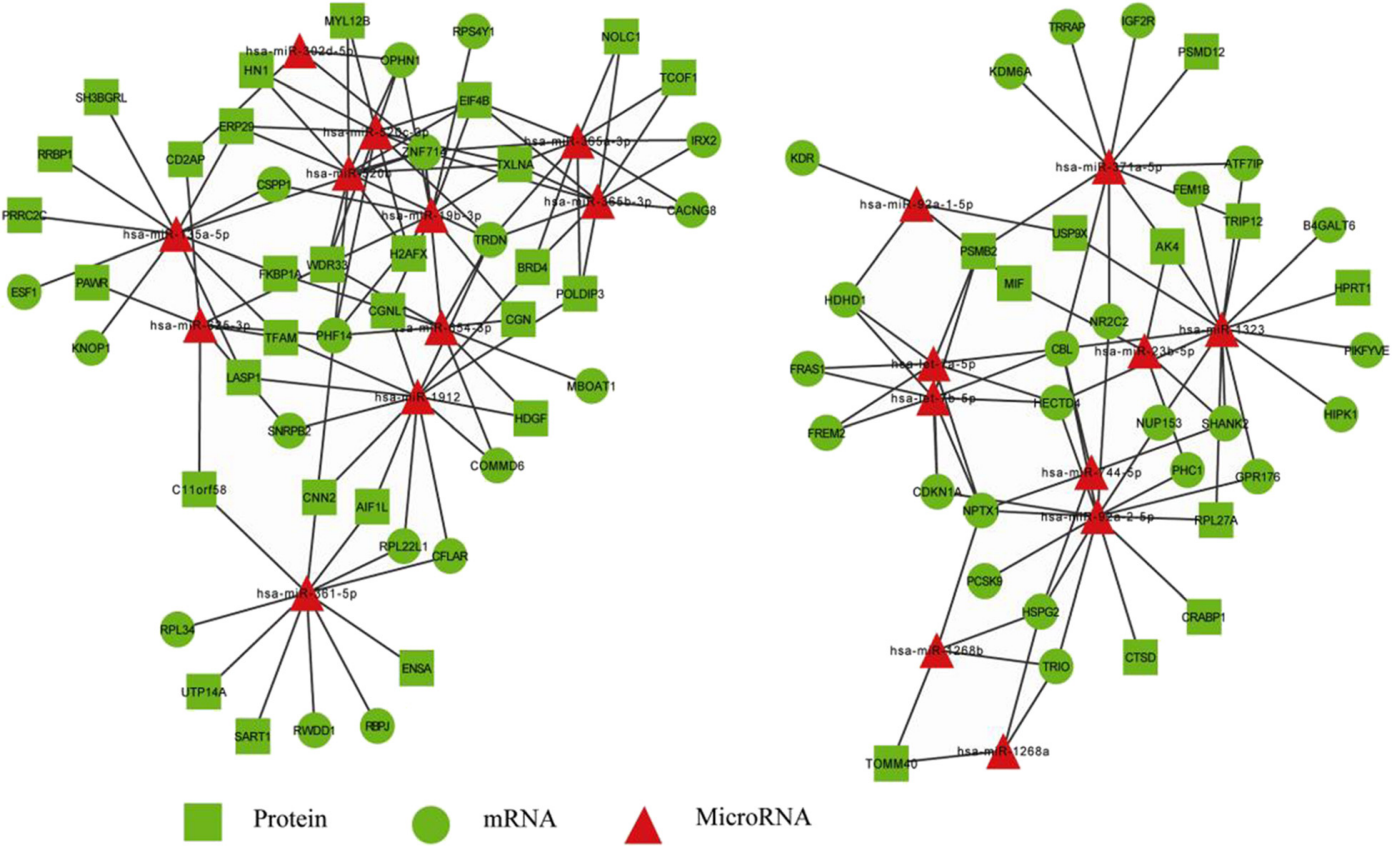
microRNA-target regulation network is composed of differentially expressed miRNAs, miRNA-target mRNAs and proteins. The mapping was made up of 39 down-regulated target proteins, 42 down-regulated mRNAs and 21 up-regulated miRNAs. Green tetragons represent down- regulated target proteins, green circles down-regulated target mRNAs, and red triangles up-regulated miRNA. The lines stand for coherent miRNAs-target proteins and miRNAs-target mRNAs interaction pairs

In order to integrate profiling datasets of differentially expressed mRNAs, microRNAs and Proteins, the mRNA-protein-microRNA regulation network was constructed with 39 down-regulated target proteins, 42 down-regulated mRNAs and 21 up-regulated miRNAs (Fig. 11). The result indicated coherent miRNAs-target proteins and miRNAs-target mRNAs interaction pairs. This demonstrates that a single miRNA can target multiple proteins and mRNAs, while a single protein or mRNA can be targeted by multiple miRNAs, which can cooperatively repress a range of targets.

## Discussion

After Takahashi and Yamanaka demonstrate that murine fibroblasts can be reprogrammed into iPSCs in 2006 [17]. The application of powerful technique to humans opened the possibility to reprogram fibroblasts and/or various specific immune cells isolated not only from healthy people but also from patients suffering of a genetic disease [6, 18]. What is more, the advances in stem cell technology of iPSCs offer the opportunity to generate and directly analyze human autoimmune disease in healthy and disease states [19]. Despite the fact that human iPSC-derived cells remained controversial to the present day, the system has attested successful in vitro replication of the main cellular characteristics already known to be associated with the modeled disease [18]. Araki et al. [20] verified that no pronounced differences in immunogenicity existed between the teratomas formed by seven iPSCs and five embryonic stem cell lines. Based on the research results, iPSCs can be derived from syngeneic autologous cells as equally as they can differentiate into specific immune cell types for modeling diseases or clinical immunotherapy [6].

Network modeling, analysis and subsequent target identification approach appear more promising in characterizing diseases [21]. Biological networks comprise of interactions between genes and proteins that co-ordinate in the regulation of cellular processes [22]. Computational and systems biological techniques can be employed to study the cellular level networks that mediate physiological system dynamics [22]. In this study, an integrative methodology was employed to combine the data obtained through multiple expression profiling methods, genomic sequence and bioinformatics analysis of in-silico target miRNA, so as to investigate the impacts of miRNA, mRNA and protein expression on SLE-iPSCs.

Differentially expressed proteins, mRNAs and miRNAs were systematically investigated. Computational methods were adopted to predict miRNA interaction and prioritize potential direct targets. Finally, mRNA-proteomic mapping, miRNA-target mRNA network and miRNA-target protein interactions were achieved. Systematic comparison results demonstrated the common physiological changes in SLE-iPSCs and NC-iPSCs. Nucleus and nucleolus in cellular component, transmembrane transport in biological process and structural constituent of ribosome in molecular function were found to be significant clustered in GO terms. That is to say, systemic lupus erythematosus (SLE) is a systemic non-organ specific autoimmune disease associated with multiple autoantibodies targeting autoantigens from the nucleus [23]. Anti-single and anti-double-stranded DNA antibodies and rheumatoid factor were marker proteins of SLE, which mainly derived from the cell nucleus. Our results showed that differentially expressed proteins, mRNAs and target miRNAs were mainly directed to nuclear constituents consistently. A subset of nuclear constituents is directed to proteins and RNAs of the ribosomes [24]. While a part of auto-antibodies directed against some antigenic site composed of a portion of both ribosomal RNA and ribosomal protein [25]. As a result, differentially expressed proteins, mRNAs and target miRNAs, which are clustered in structural constituent of ribosome, will become markers of SLE in the future.

Transport systems are essential to each living cell, which is vital for all life-endowing processes, such as communication, biosynthesis, reproduction, and cooperative behaviors [26]. Given this, SLE is viewed as expressions of combined transport dysfunction syndromes [27]. The results indicate that most differentially expressed proteins, mRNAs and target miRNAs are classified as transmembrane transport in biological process.

In the light of the central dogma, it was generally assumed that there was a direct correspondence between mRNA transcripts and generated protein expressions. By combining mRNA and protein expression data, it was found that 11 proteins and 11 RNAs were differentially expressed consistently in SLE-iPSCs and NC-iPSCs. Transgelin (TAGLN), also known as smooth muscle protein 22 (SM22), is a ubiquitous 22 kDa protein among smooth muscle tissues of normal adult vertebrates [28]. TAGLN plays a role in cell differentiation by stabilizing the cytoskeleton through actin-binding [29]. SM22/transgelin gene expression were down-regulated during a variety of cell types [30, 31], as well as in several human cancers, including lung, renal and breast cancer [30, 32, 33]. TAGLN has been proposed as a candidate tumor antigen [33]. The study results showed that TAGLN was down-regulated in SLE-iPSCs. Western blot and RT-PCR confirmed the down-regulation of both TAGLN protein and mRNA. The former belongs to transcriptional down-regulation. The mechanisms controlling the regulations of other proteins require additional study in SLE-iPSCs.

Adenylate kinase (AK) is an ATP-ADP phosphotransferase that catalyzes the interconversion of adenine nucleotides, which are involved in maintaining the homeostasis of adenine nucleotide composition in various organisms [34]. AKs are proved to be multiple isozymes, such as AK1, AK2, AK3 and AK4. These isozymes are characterized by significantly conserved sequences and their sequences are fairly comparable [35]. The gene for human AK4 was identified in 1992 [36], which is expressed mainly in tissues rich in mitochondria like the brain, heart, kidney and liver [37]. Abnormal expression of AK4 are proven in a variety of diseases, such as Parkinson's disease [38], lung tumors[39] and so on. Liu RJ et al. show that AK4 protein levels are increased in cultured cells exposed to hypoxia and in an animal model of amyotrophic lateral sclerosis (ALS), a neurodegenerative disease [40]. In our study, AK4 protein and mRNA were found to be up-regulated in SLE-iPSCs. Western blot and RT-PCR verified the up-regulation of both AK4 protein and mRNA. It can be inferred that AK4 is associated with the development of SLE. However, its mechanism needs in-depth studies with more detailed plans.

In biology process of cells, reproduction is controlled not only by protein-coding genes but also by non-coding regions, including loci that produce small RNAs [41]. MiRNAs, a family of small non-coding RNAs (typically 19–23 nucleotides) , have critical functions over a wide range of biological and pathological processes [42], such as translational repression, deadenylation, mRNA cleavage [43, 44]. MiRNAs are synthesized from short hairpin precursors and that bind to complementary messenger RNAs (mRNAs) to stimulate their degradation or repress their translation [41]. This complex circuitry of miRNA–mRNA interactions has been studied massively for over a decade in pursuit of identifying miRNA target genes and evaluating the contribution of miRNAs to the regulation of targeted genes [44]. Similarly, several studies have identified miRNA-protein interactions. Trabucchi M et al. [45] show that RNA-binding protein promotes the biogenesis of a subset of microRNAs. Davis BN [46] report the smad proteins bind a conserved RNA sequence to promote microRNA maturation by Drosha. These studies indicate proteins that directly bind different subsets of miRNA precursors and enhance their processing by either Drosha, Dicer, or both. Thus, one miRNA can target different mRNAs or proteins, and one mRNA or protein can be under the regulation of multiple miRNAs through a positive-feedback loop [44], Dimitrios G et al. found that miR-let-7 in conjunction with argonaute protein was found to bind its own primary transcript to enhance its processing by Drosha [47]. Our results indicated that miR-371a-5p, conjunct with AK4 protein, was involved in SLE.

The aim of presenting this report is to help predict miRNA-RNA targets through the integrated analysis of miRNA and mRNA expression profiling, as well as miRNA protein targets using miRNA and protein regulated network. The miRNA level of miR-371a-5p and let-7a-5p were down-regulated in SLE-iPSC and verified using Q-PCR. The up-regulation of AK4 involved in nucleotide biosynthesis may suggest a general acceleration of anabolic metabolism induced by down-regulated miR-371a-5p, which may contribute to SLE. Recent studies demonstrated that miR-371-5p was significantly elevated in gastric cancer (GC) patients and regulated hepatocellular carcinoma (HCC) cell proliferation, the miR-371-5 cluster has proved to be a novel prognostic factor and therapeutic target for tumors and disease [48, 49]. Let-7a is highly conserved among species in sequence and function [50], which operates as a tumor suppressor in human cells [51–53]. Serguienko A et al. [51] show that let-7a down-regulates key anabolic enzymes and increases both oxidative phosphorylation and glycolysis in triple-negative breast cancer and metastatic melanoma cell lines. Moreover, let-7a causes mitochondrial ROS production concomitant with the up-regulation of oxidative stress responsive genes. Loss or down-regulation of let-7 levels is associated with increased cancer aggressiveness and poor clinical outcome [54]. This report also indicated that let-7a-5p was down-regulated, which may be involved in SLE development.

## Conclusion

In summary, a range of production SLE-iPSCs has been analyzed to investigate the regulatory mechanism. In order to integrate the biological system, global miRNA, mRNA and proteomic expression profiling were employed in parallel. 1099 mRNAs, 223 microRNAs and 94 differentially expressed proteins were verified. Computational and systems biological techniques can be employed to study the regulated networks that mediate physiological system dynamics. To investigate the influence of miRNA on these processes, potential targets of differentially expressed mRNAs and Proteins were predicted using miRanda, TargetScan and Pictar. Gene ontology (GO) analysis of differentially expressed mRNAs and proteins found that identified biological processes drive proliferation including mRNA processing, translation and so on. Western blot and RT-PCR confirmed the up-regulation of AK4 protein and mRNA, as well as the down-regulation of TAGLN protein and mRNA levels in SLE-iPSCs. MiR-371a- 5p and let-7a-5p were down-regulated and verified using RT-PCR. The up-regulation of AK4 involved in nucleotide biosynthesis suggests a general acceleration of anabolic metabolism induced by down-regulated miR-371a-5p, which may contribute to SLE. It is believed that the integration of multiple profiling datasets will provide a useful tool for biologists who are investigating biological networks and seeking to identify direct regulators of mRNAs and proteins. It is also expected that the knowledge gained from this study can assess the usefulness of the pathogenesis and novel biomarker candidates of SLE, which may develop a new way for diagnosis of SLE.

## Methods

All studies and other procedures were approved by the ethics committee of the Shenzhen People’s Hospital (Shenzhen, China) or the Guangzhou Institutes of Biomedicine and Health (Guangzhou, China). 4 patients (four women, mean age 39, range 30–46) were diagnosed as active SLE with SLEDAI > 8. Patients treated with immunosuppressant within 3 months were excluded. Equivalent subjects, age and sex matched, was recruited as healthy controls. All participating subjects were explained their participation rights. Written informed consent was obtained. Renal tubular cells from urine of participating subjects were reprogrammed to generate human iPSCs clone. One SLE-iPSC clone and control-iPSC clone were identified [9], and which morphology were identified[9]. All of the samples were collected and immediately frozen in liquid nitrogen and stored at −80 °C.

### Sequencing of mRNA and miRNA

Total RNA was extracted from SLE-iPSCs and Control-iPSCs using Trizol (Invitrogen, Carlsbad, USA) according to the manufacturer's instructions. The concentration and quality of total RNA were measured by the UV absorbance at 260 nm and 280 nm (A260/280) and checked by gel electrophoresis. The polyA mRNAs were selected by RNA Purification Beads (Illumina, SanDiego, CA). MicroRNA isolation was carried out from the total RNA using mirVana™ microRNA isolation kit (Ambion, Austin, TX) according to the manufacturer's instructions. Total mRNA and miRNA isolated from three independent cultures were, respectively, pooled for subsequent library construction and sequence analysis. The libraries of mRNA and miRNA were constructed by using the Illumina TruSeq RNA Sample Prep Kit v2-Set A and TruSeq Small RNA Sample Prep Kit Set A, respectively, sequenced by the Illumina HiSeq™ 2000 System. Adaptor sequences were subsequently trimmed to clean full-length reads and formatted into a non-redundant FASTQ format. The high-quality clean reads were mapped to the reference human genome using the SOAP (version 2.0) software. Sequences that perfectly matched the genome along their entire length were considered for next analyses. Differential expression of mRNA and miRNA between SLE-iPSCs and Control-iPSCs were calculated by relative expression analysis.

### Protein extraction, identification and relative quantification

The process of protein extraction and iTRAQ sample labeling were performed as described by our previous research [55]. In briefly, the iPSC cells were harvested and washed with ice-cold PBS, then lysed in ice-cold lysis buffer. Protein concentration was determined by Bradford Protein Assay Kit (Pierce). One hundred micrograms of protein from each corresponding group was digested with trypsin, and labeled according to the iTRAQ protocol (Applied Biosystems). The labeled digests were subjected to liquid chromatography coupled with tandem mass spectrometry (LC-MS/MS) analysis. Once dates had been acquired, all data files were processed by Mascot (version 2.3.02) and searched against the IPI_human v3.87 database for peptide identification and quantification, peptide grouping into proteins and protein ratio calculation. Only proteins with ≥ 1 peptides matched a ≥ 1 fold difference in abundance in both directions and a p-value < 0.05 were considered to be differentially expressed protein.

### MicroRNA target predictions from differentially expressed mRNA and differentially expressed protein

MiRNA target prediction was performed using three software: PicTar: http://pictar.mdc-berlin.de/; miRanda v5: http://www.ebi.ac.uk/enright%20srv/microcosm/htdocs/targets/v5/; TargetScan 6.2: http://www.targetscan.org/vert/. When predicted at least by two software at the same time, the result was considered reliable. Candidate miRNA-target pairs were collected and further analyzed.

### Data integration and analysis

The experiment, identified differentially expressed protein, mRNA and miRNA, was combined with microRNA target predictions to form integrated regulatory networks. Namely, those data used as the input for differential expression identification and miRNA target prediction. Finally, acquired data were analyzed with the KEGGSOAP software (http://www.bioconductor.org/packages/2.4/bioc/html/KEGG SOAP.html). The results of regulatory networks were handled in cytoscape software (http:// cytoscape. org).

### Gene ontology

The predicted miRNA target mRNA, protein and the differentially expressed mRNA, proteins were subjected to the gene ontology analysis using KEGGSOAP software. The target predictions and differentially expressed dates were mapped to the GO annotation dataset, and the enriched cellular component (CC), biological process (BP) and molecular function (MF) were extracted using the hypergeometric test. a P < 0.05 was considered to be significant.

### Western blotting

The SLE-iPSCs and NC-iPSCs were lysed with ice-cold lysis buffer. Proteins were separated by SDS/PAGE, electrophoresed, and transferred onto a PVDF membrane. The membrane was incubated with primary antibodies to anti-TAGLN 1 : 500 (GeneTex) or AK4 1 : 2000 (abcam) at 4 °C overnight, followed by horseradish peroxidase (HRP)-conjugated goat anti-rabbit immunoglobulin G (IgG) 1 : 2000 (southern biotech) at room temperature for 2 h. The antibody-bound proteins were analyzed by using immobilon western chemilum HRP substrate (Millipore). The quantitative analysis of the immunoblot results were performed using Image J software.

### Q-PCR analysis

Total RNA of SLE-iPSCs and NC-iPSCs was extracted with trizol (Invitrogen) and reversed to cDNA using iScript™ cDNA Synthesis Kit (Bio-Rad). The following specific primers were used: TAGLN, forward: 5’-ATGGCGTGATTCTGAGCAA-3’, reverse: 5’-ATCTGCTTGAAGACCATGG A-3’; AK4, forward: 5’-GCCCAGGCTAATCTATGAAG-3’, reverse: 5’-CAAGGAGCTCAAAAG CCTAT-3’; 18 s rRNA, forward: 5’-CCTGGATACCGCAGCTAGGA-3’, reverse: 5’-GCGGCGCAATACGAATGCC CC-3’; miR-371a-5p, forward: 5’-ACACTCCAGCTGGGTAGCTTATCAGACTGAT G-3’; let-7a-5p, forward: 5’-ACA CTCCAGCTGGGACTCAAACTGTGGGGGC-3’, miRNA, reverse: 5’-CTCAACT GGTGTCGTGGA-3’; U6, forward: 5’-CTCGCTTCGGCAGCACA-3’, reverse: 5’-AA CGCTTCACG AATTT GCGT-3’. Q-PCR was performed on a Real-time PCR system (Bio-Rad) using individual PCR tubes (Bio-Rad). The relative expression level was calculated from a relative standard curve obtained by using log dilutions of cDNA containing genes of TAGLN, AK4, miR-371a-5p, let-7a-5p. The relative expression was quantified using the “2^-ΔΔCt^” method [56]. The average of three independent analyses for each gene was calculated.

## Additional files

Additional file 1:
**Table S1.** Differentially expressed mRNA. (XLSX 271 kb) Additional file 2:
**Table S2.** Differentially expressed microRNA. (XLSX 22 kb) Additional file 3:
**Table S3.** Up-regulation of differentially expressed proteins. (XLSX 11 kb) Additional file 4:
**Table S4.** Down-regulation of differentially expressed proteins. (XLSX 15 kb) Additional file 5:
**Table S5.** KEGG analysis of differentially expressed mRNA. (XLSX 28 kb) Additional file 6:
**Table S6.** KEGG analysis of differentially expressed protein. (XLSX 16 kb)

## References

[1] Perl A (2010). Pathogenic mechanisms in systemic lupus erythematosus. Autoimmunity.

[2] de Macedo PA, Borba EF, Viana VDT, Leon EP, Testagrossa LD, Barros RT, Nascimento AP, Bonfa E (2011). Antibodies to ribosomal P proteins in lupus nephritis: a surrogate marker for a better renal survival?. Autoimmun Rev.

[3] Herbst R, Liu Z, Jallal B, Yao YH (2012). Biomarkers for systemic lupus erythematosus. Int J Rheum Dis.

[4] Quan JX, Lakhanpal A, Reddy MM, Zaman S, Li QZ, German DC, Olsen NJ, Kodadek T, Karp DR (2014). Discovery of biomarkers for systemic lupus erythematosus using a library of synthetic autoantigen surrogates. J Immunol Methods.

[5] Abulaban KM, Brunner HI (2015). Biomarkers for Childhood-Onset Systemic Lupus Erythematosus. Curr Rheumatol Rep.

[6] Jiang ZP, Han YM, Cao XT (2014). Induced pluripotent stem cell (iPSCs) and their application in immunotherapy. Cell Mol Immunol.

[7] Guan X, Mack DL, Moreno CM, Strande JL, Mathieu J, Shi YG, Markert CD, Wang ZJ, Liu GH, Lawlor MW (2014). Dystrophin-deficient cardiomyocytes derived from human urine: New biologic reagents for drug discovery. Stem Cell Res.

[8] Thatava T, Armstrong AS, De Lamo JG, Edukulla R, Khan YK, Sakuma T, Ohmine S, Sundsbak JL, Harris PC, Kudva YC, et al. Successful disease-specific induced pluripotent stem cell generation from patients with kidney transplantation. Stem Cell Res Therapy. 2011;2. 10.1186/scrt89 PMC3340557 22142803

[9] Chen YY, Luo RP, Xu Y, Cai XJ, Li WX, Tan KB, Huang JR, Dai Y (2013). Generation of systemic lupus erythematosus-specific induced pluripotent stem cells from urine. Rheumatol Int.

[10] Yamana R, Iwasaki M, Wakabayashi M, Nakagawa M, Yamanaka S, Ishihama Y (2013). Rapid and deep profiling of human induced pluripotent stem cell proteome by one-shot NanoLC-MS/MS analysis with meter-scale monolithic silica columns. J Proteome Res.

[11] Plath K, Lowry WE (2011). Progress in understanding reprogramming to the induced pluripotent state. Nat. Rev. Genet.

[12] Chang G, Gao S, Hou XF, Xu ZJ, Liu YF, Kang L, Tao Y, Liu WQ, Huang B, Kou XC (2014). High-throughput sequencing reveals the disruption of methylation of imprinted gene in induced pluripotent stem cells. Cell Res.

[13] Bartel DP (2004). MicroRNAs: Genomics, biogenesis, mechanism, and function. Cell.

[14] Wang YP, Li KB (2009). Correlation of expression profiles between microRNAs and mRNA targets using NCI-60 data. BMC Genom.

[15] Cheng C, Li LM (2008). Inferring MicroRNA Activities by Combining Gene Expression with MicroRNA Target Prediction. PloS One.

[16] Gerstberger S, Hafner M, Tuschl T (2014). A census of human RNA-binding proteins. Nat Rev Genet.

[17] Takahashi K, Yamanaka S (2006). Induction of pluripotent stem cells from mouse embryonic and adult fibroblast cultures by defined factors. Cell.

[18] Bui YK, Cordes KR, Hastie S, Srivastava D (2012). Cardiac Disease Modeling of Familial Left Ventricular Noncompaction Cardiomyopathy Using Induced Pluripotent Stem Cell. Circulation.

[19] Pessach IM, Ordovas-Montanes J, Zhang SY, Casanova JL, Giliani S, Gennery AR, Al-Herz W, Manos PD, Schlaeger TM, Park IH (2011). Induced pluripotent stem cells: A novel frontier in the study of human primary immunodeficiencies. J Allergy Clin Immun.

[20] Araki R, Uda M, Hoki Y, Sunayama M, Nakamura M, Ando S, Sugiura M, Ideno H, Shimada A, Nifuji A (2013). Negligible immunogenicity of terminally differentiated cells derived from induced pluripotent or embryonic stem cells. Nature.

[21] Mardinoglu A, Nielsen J (2012). Systems medicine and metabolic modelling. J Internal Med.

[22] Somvanshi PR, Venkatesh KV (2014). A conceptual review on systems biology in health and diseases: from biological networks to modern therapeutics. Syst Synth Biol.

[23] De Bandt M (2006). Lessons for lupus from tumour necrosis factor blockade. Lupus.

[24] Anderson CJ, Neas BR, Uchiumi T, Stafford HA (2001). Autoantibodies to the 20-kDa ribosomal proteins: identification, characterization, and new aspects on prevalence in systemic Lupus erythematosus. Clin Immunol.

[25] Sturgill BC, Carpenter RR (1965). Antibody to Ribosomes in Systemic Lupus Erythematosus. Arthritis Rheum.

[26] Yen MR, Choi J, Saier MH (2009). Bioinformatic Analyses of Transmembrane Transport: Novel Software for Deducing Protein Phylogeny, Topology, and Evolution. J Mol Microb Biotech.

[27] Rudin DO (1981). The choroid plexus and system disease in mental illness. II. Systemic lupus erythematosus: a combined transport dysfunction model for schizophrenia. Biol Psychiat.

[28] Lees-Miller JP, Heeley DH, Smillie LB, Kay CM (1987). Isolation and characterization of an abundant and novel 22-kDa protein (SM22) from chicken gizzard smooth muscle. J. Biol. Chem.

[29] Camoretti-Mercado B, Forsythe SM, LeBeau MM, Espinosa R, Vieira JE, Halayko AJ, Willadsen S, Kurtz B, Ober C, Evans GA (1998). Expression and cytogenetic localization of the human SM22 gene (TAGLN). Genomics.

[30] Shields JM, Rogers-Graham K, Der CJ (2002). Loss of transgelin in breast and colon tumors and in RIE-1 cells by Ras deregulation of gene expression through Raf-independent pathways. J. Biol. Chem.

[31] Bregant E, Renzone G, Lonigro R, Passon N, Di Loreto C, Pandolfi M, Scaloni A, Tell G, Damante G (2009). Down-regulation of SM22/transgelin gene expression during H9c2 cells differentiation. Mol. Cell. Biochem.

[32] Li LS, Kim H, Rhee H, Kim SH, Shin DH, Chung KY, Park KS, Paik YK, Chang J, Kim H (2004). Proteomic analysis distinguishes basaloid carcinoma as a distinct subtype of nonsmall cell lung carcinoma. Proteomics.

[33] Klade CS, Voss T, Krystek E, Ahorn H, Zatloukal K, Pummer K, Adolf GR (2001). Identification of tumor antigens in renal cell carcinoma by serological proteome analysis. Proteomics.

[34] Yoneda T, Sato M, Maeda M, Takagi H (1998). Identification of a novel adenylate kinase system in the brain: cloning of the fourth adenylate kinase. Brain Res. Mol. Brain Res.

[35] Schulz GE, Schiltz E, Tomasselli AG, Frank R, Brune M, Wittinghofer A, Schirmer RH (1986). Structural relationships in the adenylate kinase family. Eur J Biochem.

[36] Xu G, O'Connell P, Stevens J, White R (1992). Characterization of human adenylate kinase 3 (AK3) cDNA and mapping of the AK3 pseudogene to an intron of the NF1 gene. Genomics.

[37] Panayiotou C, Solaroli N, Karlsson A (2014). The many isoforms of human adenylate kinases. Int J Biochem Cell B.

[38] Garcia-Esparcia P, Hernandez-Ortega K, Ansoleaga B, Carmona M, Ferrer I. Purine metabolism gene deregulation in Parkinson's disease. Neuropathol Appl Neurobiol. 2015;41(7):926-40. 10.1111/nan.12221 25597950

[39] Greengard O, Head JF, Goldberg SL (1980). Uridine kinase, adenylate kinase, and guanase in human lung tumors. Cancer Res.

[40] Liu RJ, Strom AL, Zhai JJ, Gal J, Bao SL, Gong WM, Zhu HN (2009). Enzymatically inactive adenylate kinase 4 interacts with mitochondrial ADP/ATP translocase. Int J Biochem Cell B.

[41] Zhuang X, Li ZM, Lin HN, Gu L, Lin Q, Lu ZX, Tzeng CM (2015). Integrated miRNA and mRNA expression profiling to identify mRNA targets of dysregulated miRNAs in non-obstructive azoospermia. Sci Rep-Uk.

[42] He ZP, Kokkinaki M, Pant D, Gallicano GI, Dym M (2009). Small RNA molecules in the regulation of spermatogenesis. Reproduction.

[43] Wilczynska A, Bushell M (2015). The complexity of miRNA-mediated repression. Cell Death Differ.

[44] Bak RO, Mikkelsen JG (2014). miRNA sponges: soaking up miRNAs for regulation of gene expression. Wiley Interdisciplinary Rev RNA.

[45] Trabucchi M, Briata P, Garcia-Mayoral M, Haase AD, Filipowicz W, Ramos A, Gherzi R, Rosenfeld MG (2009). The RNA-binding protein KSRP promotes the biogenesis of a subset of microRNAs. Nature.

[46] Davis BN, Hilyard AC, Nguyen PH, Lagna G, Hata A (2010). Smad Proteins Bind a Conserved RNA Sequence to Promote MicroRNA Maturation by Drosha. Molecul Cell.

[47] Zisoulis DG, Kai ZS, Chang RK, Pasquinelli AE (2012). Autoregulation of microRNA biogenesis by let-7 and Argonaute. Nature.

[48] He D, Miao HL, Xu YM, Xiong LH, Wang Y, Xiang HX, Zhang H, Zhang ZY (2014). MiR-371-5p facilitates pancreatic cancer cell proliferation and decreases patient surviva. PloS One.

[49] Liu RY, Diao CF, Zhang Y, Wu N, Wan HY, Nong XY, Liu M, Tang H (2013). miR-371-5p down-regulates pre mRNA processing factor 4 homolog B (PRPF4B) and facilitates the G1/S transition in human hepatocellular carcinoma cells. Cancer Lett.

[50] Pasquinelli AE, Reinhart BJ, Slack F, Martindale MQ, Kuroda MI, Maller B, Hayward DC, Ball EE, Degnan B, Muller P (2000). Conservation of the sequence and temporal expression of let-7 heterochronic regulatory RNA. Nature.

[51] Serguienko A, Grad I, Wennerstrom AB, Meza-Zepeda LA, Thiede B, Stratford EW, Myklebost O, Munthe E (2015). Metabolic reprogramming of metastatic breast cancer and melanoma by let-7a microRNA. Oncotarget.

[52] Liu YC, Yin BD, Zhang CC, Zhou LB, Fan J (2012). Hsa-let-7a functions as a tumor suppressor in renal cell carcinoma cell lines by targeting c-myc. Biochem Bioph Res Co.

[53] Guled M, Lahti L, Lindholm PM, Salmenkivi K, Bagwan I, Nicholson AG, Knuutila S (2009). CDKN2A, NF2, and JUN Are Dysregulated Among Other Genes by miRNAs in Malignant Mesothelioma-A miRNA Microarray Analysis. Gene Chromosome Canc.

[54] Nair VS, Maeda LS, Ioannidis JPA (2012). Clinical Outcome Prediction by MicroRNAs in Human Cancer: A Systematic Review. J. Natl. Cancer Inst..

[55] Lan P, Li WF, Wen TN, Shiau JY, Wu YC, Lin WD, Schmidt W (2011). iTRAQ Protein Profile Analysis of Arabidopsis Roots Reveals New Aspects Critical for Iron Homeostasis. Plant Physiol.

[56] Schmittgen TD, Livak KJ (2008). Analyzing real-time PCR data by the comparative C(T) method. Nat Protoc.

